# Assessment and indicators of kinematic behavior and perceived fatigability

**DOI:** 10.1590/1806-9282.20230924

**Published:** 2024-02-26

**Authors:** Helena Silva-Migueis, Eva María Martínez-Jiménez, Israel Casado-Hernández, Adriano Dias, Ana Júlia Monteiro, Rodrigo Brandão Martins, João Marcos Bernardes, Daniel López-López, Juan Gómez-Salgado

**Affiliations:** 1 University of A Coruña, Faculty of Nursing and Podiatry, Industrial Campus of Ferrol, Research, Health and Podiatry Group, Department of Health Sciences - Ferrol, Spain.; 2 Escola Superior de Saúde da Cruz Vermelha Portuguesa-Lisboa, Department of Physiotherapy - Lisbon, Portugal.; 3 Complutense University of Madrid, Faculty of Nursing, Physiotherapy and Podiatry - Madrid, Spain.; 4 Universidade Estadual Paulista, Department of Public Health, Graduate Program in Collective/Public Health, Botucatu Medical School - Botucatu (SP), Brazil.; 5 University of Huelva, Faculty of Labour Sciences, Department of Sociology, Social Work and Public Health - Huelva, Spain.; 6 Espíritu Santo University, Safety and Health Postgraduate Programme - Guayaquil, Ecuador.

**Keywords:** Fatigue, Aged, Upper extremity, Exercise therapy, Accelerometry, Symptom assessment

## Abstract

**OBJECTIVE:**

The objective of this study was to investigate the relationship between upper limb kinetics and perceived fatigability in elderly individuals during an upper limb position sustained isometric task.

**METHODS:**

A total of 31 elderly participants, 16 men (72.94±4.49 years) and 15 women (72.27±6.05 years), performed a upper limb position sustained isometric task. Upper-limb acceleration was measured using an inertial measurement unit. Perceived fatigability was measured using the Borg CR10 scale.

**RESULTS:**

Higher mean acceleration in the x-axis throughout the activity was associated with higher final perceived fatigability scores. Moderate correlations were observed between perceived fatigability variation and mean acceleration cutoffs in all axes during the second half of the activity. In women, significant correlations were found between all perceived fatigability cutoffs and mean acceleration in the y- and x-axes. However, in men, the relationships between perceived fatigability variation and mean acceleration were more extensive and stronger.

**CONCLUSION:**

The acceleration pattern of the upper limb is linked to perceived fatigability scores and variation, with differences between sexes. Monitoring upper limb acceleration using a single inertial measurement unit can be a useful and straightforward method for identifying individuals who may be at risk of experiencing high perceived fatigability or task failure.

## INTRODUCTION

Perceived fatigability (PcFat) refers to the subjective sensation of weariness, increasing effort, and a mismatch between effort expended and actual performance in the context of a specific activity. It reflects the changes in sensations that regulate an individual’s well-being and depends on both the physiological capacity of the body to maintain homeostasis and the individual’s psychological state^1^.

The determinants of perceived motor fatigability can be categorized into three dimensions: (a) the perceptual-discriminatory dimension; (b) the affective-motivational dimension; and (c) the cognitive-evaluative dimension. The three dimensions of this framework interact with each other^2^.

However, performance fatigability is described as a “decline in an objective measure of physical performance over a discrete period.” It is influenced by muscle contractile function and the central nervous system’s ability to meet the demands of a task^1^.

As dimensions of fatigue, the interdependence between performance and PcFat is highlighted and both contribute to the overall experience of fatigue that can be modulated by different factors, including age, sex, presence of diseases, level of fitness, and specific characteristics of the tasks being performed^3^.

In elderly individuals, fatigue’s prevalence is high and can significantly impact their ability to perform activities of daily living^4^, reduce social interaction^5^, and predict later health services usage^6^ and mortality^7^.

Even in the absence of disability, aged skeletal muscle is expected to become slower and weaker, reveals a powerful decrease in the efficiency of voluntary contractions, as well as is less stable in the course of the efficiency of isometric contractions, especially at low force rates^8^.

Particularly, upper-limb position-sustained tasks impose additional ventilatory and postural loads on the thoracic complex^9^, leading to modifications in breathing patterns and upper-limb kinematics^10^.

Isometric activity, such as position-sustained tasks, can play a crucial role in recreational, sports, and rehabilitation plans, especially in the aging population, since isometric activities offer benefits such as improved joint stability, lower blood pressure, and reduced overall pain^11^.

Since fatigability can also manifest as decreased movement accuracy, impaired proprioception acuity, and reduced co-contraction during precision movements^12^, a biomechanical approach can help identify fatigue-related changes in movement patterns over time^13^.

Up to now, the study of kinematic changes caused by fatigue involves optoelectronic or equivalent motion-capture systems. However, inertial measurement units (IMUs) have emerged as nonintrusive and portable devices for kinematic assessment. They combine information from accelerometers, gyroscopes, and magnetometers to provide continuous and accurate orientation output for real-time applications and daily-life environments^14^. Their validity and inter-system agreement have been demonstrated^15^.

Considering this, understanding the relationship between upper limb kinematics and PcFat during a position-sustained isometric task is essential to understand if and how the kinematic behavior of the upper limb is related to the subjective experience of fatigue.

Therefore, this study aims to investigate the relationship between PcFat and upper limb kinematic behavior during an upper limb position-sustained isometric task (ULPSIT), according to the hypothesis that PcFat evolution is associated with upper limb acceleration throughout the activity duration.

## METHODS

### Design and sample

A quasi-experimental study was conducted in accordance with the protocol approved by the ESSCVP-Lisboa Ethics Committee, Portugal, and registered at ClinicalTrials.gov (NCT04938791).

The study comprised 31 elderly participants (72.61±5.23 years old), 16 men (72.94±4.49 years old) and 15 women (72.27±6.05 years old), who resided in the community. To be eligible for inclusion in the study, participants had to be apparently healthy and above 65 years of age. However, individuals with the following conditions were excluded^16^: (1) history of cardiovascular and/or respiratory disease, hypertension, and exercise intolerance, (2) cognitive or neurological disorders, (3) body mass index≥40, and (4) neuromuscular or orthopedic disorder.

### Inertial measurement unit and perceived fatigability

The three-dimensional (3D) acceleration of the upper limb was captured using one inertial measurement unit (IMU) (MTw Awinda, the Netherlands). The orientation of the IMU was computed using the Xsens Kalman Filter for a 3 degrees-of-freedom (3DoF) orientation known as human motion (XKF3hm).

These IMUs (MTw units) utilize advanced signal processing techniques to handle raw data and incorporate StrapDown Integration (SDI) algorithms^17^. These units transmit the data wirelessly through an Awinda Station (Xsens Technologies B.V., Enschede, the Netherlands) to a recording personal computer (PC). The data were recorded at a sampling rate of 100 Hz, ensuring precise sampling even though the units have the capability to sample at rates higher than 1 kHz.

For data visualization and recording, the MT Manager software version 4.4.0 (Xsens, Enschede, the Netherlands) was employed.

The activity-related PcFat was evaluated using the Portuguese Borg 10-point category-ratio scale (Borg CR10 Scale^®^). This scale is a widely recognized general intensity scale with category-ratio properties and is utilized to assess subjective sensations of exertion, including local fatigue, breathlessness, dyspnea, discomfort, and pain.

### Research procedure

Initially, subjects were weighed and measured, and they were then asked to complete a brief survey to provide additional personal information. Following the questionnaire, participants were given a 5-min rest period in a comfortable chair.

Furthermore, the positioning of the IMU was performed. The IMU was placed on the external side of the humerus of the dominant arm (right arm). Its reference coordinate system was configured to have the x-axis pointing forward, the y-axis pointing upward, and the z-axis pointing laterally, perpendicular to the sagittal plane. Double-sided tape was used to minimize any soft tissue artifacts^18^.

Following the preparatory procedures, participants were instructed to perform a specific task involving the flexion of their upper arm until it reached a 90^°^ angle, with their hands facing each other, similar to the position used in a previous study^19^. They were instructed to maintain this posture for as long as possible.

### Data processing and statistical analysis

The demographic and anthropometric variables of the sample and all variables of the study were analyzed. The mean, standard deviation (SD), and maximum and minimum values of these variables were calculated.

Either the Student’s t-test for independent samples (a parametric test suitable for normal distribution) or the Mann-Whitney U test (a counterpart test for nonparametric distribution) was used, depending on the nature of the variable. The Shapiro-Wilk normality test with a significance level set at p>0.05 was used for decision-making. The Levene’s test was conducted to assess the equality of variances.

Spearman’s correlation coefficient was calculated to measure the strength and direction of the association between all variables.

The statistical analyses were performed using the SPSS statistical software, version 28.0. The significance level for all tests was set at p<0.05, with a confidence interval of 95%.

## RESULTS

### Sample characterization

#### Demographic and anthropometric characteristics

A detailed overview of the participants’ demographic and anthropometric characteristics is displayed in Table 1.

**Table 1.** Sample’s demographic and anthropometric characteristics.

Sample characteristics	Total sample (n=31) Mean±SD (range)	Women (n=15) Mean±SD (range)	Men (n=16) Mean±SD (range)	p-value^1^	Effect size (Cohen´s d)
Age (years)	72.61±5.23	72.27±6.05	72.94±4.49	0.727	0.126
	(65-85)	(65-85)	(65-82)		
Weight (kg)	73.18±13.01	67.57±12.37	78.44±11.61	**0.017**	0.907
	(48-100)	(48-86.5)	(57.90-100)		
Height (m)	1.60±0.08	1.54±0.43	1.65±0.06	**<0.001**	2.116
	(1.48-1.76)	(1.48-1.64)	(1.57-1.76)		
BMI (kg/m^2^)	28.71±4.66	28.69±5.30	28.74±4.15	0.980	0.009
	(21.57-37.94)	(21.57-37.94)	(23.42-35.61)		

In all analyses, p<0.05 was considered statistically significant. ^1^Independent-samples Student’s t-test. Abbreviations: BMI: body mass index; SD: standard deviation.

#### Sample’s perceived fatigability status characterization

Table 2 provides data on two distinct statuses of PcFat: the variation of PcFat in the first half of the activity (∆PcFat 0-50%) and the second half of the activity (∆PcFat 50-75%).

**Table 2.** Sample’s fatigability characterization in different periods.

Sample characteristics	Total sample Mean±SD (range)	p-value	Effect size (Cohen´s d)	Women Mean±SD (range)	Men Mean±SD (range)	p-value	Effect size (Cohen´s d)
PcFat strong (%TTF)	66.95±22.61	_______	_______	74.74±19.89	59.65±23.13	0.0621	0.698
	(20.79-100.00)			(38.79-100.00)	(20.79-100.00)		
∆PcFat 0-50%	3.18±2.15	**0.016**	0.306	2.57±1.41	3.75±2.59	0.129^1^	0.562
	(0.00-8.00)			(0.00-5.00)	(0.00-8.00)		
∆PcFat 50-100%	4.53±1.99			4.63±2.28	4.43±1.74	0.789^1^	0.097
	(1.00-9.50)			(1.00-9.50)	(1.00-7.00)		
Weak PcFat (s)	284.58±168.42	**0.004**	0.368	258.69±163.83	216.07±174.28	0.232^2^	0.297
	(120.00-840.00)			(120.00-780.00)	(180.00-840.00)		
Strong PcFat (s)	189.94±224.19			111.00±100.89	263.98±280.99	0.060^2^	0.715
	(0.00-1143.00)			(0.00-284.00)	(0.00-1143.00)		

In all analyses, p<0.05 was considered statistically significant. ^1^Independent-samples Student’s t-test. ^2^Independent-samples Mann-Whitney U test. Abbreviations: SD: standard deviation; PcFat, perceived fatigability; TTF, time to task failure.

### Upper limb acceleration in the xx-, y-, and zz -axes and perceived fatigability

The results show a positive and statistically significant relationship between PcFat scores at 75 and 100% cutoffs and x-axis mean acceleration (mA). Early relationships were also detected between PcFat scores and y-axis mA in women, but no correlations were found in men. The correlation analysis is summarized in Table 3.

**Table 3.** Relationship between acceleration in the x-, y-, and z-axes and perceived fatigability scores in the total sample and in women^1^.

	Total sample	Women
PcFat 25%	___	Y10% (p=0.596*, 95%CI [0.103, 0.853])
		Y20% (p=0.555*, 95%CI [0.044, 0.836])
PcFat 50%	___	Y10% (p= 0.543*, 95%CI [0.026, 0.831])
PcFat 75%		X50% (p=0.535*, 95%CI [0.014, 0.827])
		X60% (p=0.542*, 95%CI 0.024, 0.827])
		X70% (p=0.628*, 95%CI [0.154, 0.867])
	X100% (p=0.377*, 95%CI [0.015, 0.651])	X80% (p=0.644**, 95%CI [0.180, 0.873])
		X90% (p=0.673**, 95%CI [0.229, 0.885])
		X100% (p=0.544*, 95%CI [0.027, 0.831])
PcFat 100%	X40% (p=0.379*, 95%CI [0.170, 0.653])	X50% (p=0.525*, 95%CI [0.000, 0.823])
	X50% (p=0.395*, 95%CI [0.036, 0.663])	X60% (p=0.563*, 95%CI [0.055, 0.840])
	X60% (p=0.429*, 95%CI [0.077, 0.686])	X70% (p=0.589*, 95%CI [0.093, 0.851])
	X70% (p=0.454*, 95%CI [0.108, 0.702])	X80% (p=0.518*, 95%CI [-0.010, 0.820])
	X80% (p=0.415*, 95%CI [0.061, 0.677])	X90% (p=0.591*, 95%CI [0.096, 0.851])
	X90% (p=0.441*, 95%CI [0.091, 0.693])	X100% (p=0.675**, 95%CI [0.233, 0.886])
	X100% (p=0.504**, 95%CI [0.171, 0.733])	

*Statistically significant correlation, p<0.05 (two-tailed); **Statistically significant correlation, p<0.01 (two-tailed). Abbreviations: X%, acceleration in x-axis at a determined % of activity time; PcFat%, perceived fatigability at a determined % of activity time. ^1^No correlations were detected for men.

## DISCUSSION

Our previous research showed a positive relationship between perceived and performance fatigability in the final stage of the activity differences and significant changes in mA, which revealed the presence of upper limb motion in the sagittal plane and an overall increase in movement variability, with sex-related differences^20^ ^,^ ^21^.

This study aimed to examine the relationship between PcFat and upper limb kinematic behavior during a ULPSIT.

Our findings confirmed the hypothesis, revealing specific relationships between PcFat and its variation with mA acceleration on different axes, and also highlighted sex-related differences in kinematic behavior and their relationship with PcFat in women and with PcFat variation in men.

PcFat during physical activity depends on the psychophysiological state of an individual, which influences perceptual, affective, and cognitive processes during activity, and is influenced by many adjustments that occur in the modulating factors, reflecting changes in the sensations that serve as a mechanism for regulating performance and performer integrity^1^.

Changes in muscle recruitment occur with modifications in muscle synergies because the role of a fatigued muscle within a muscle synergy structure may change, producing adaptations in the recruitment of the remaining muscles in the synergy structure^22^, and also in co-contraction (agonist-antagonist)^23^.

The ULPSIT may have produced alterations in muscular activation and recruitment, which should be explored in future studies, and induced upper limb acceleration changes that may have influenced the way women and men sense fatigue and how they respond to it^3^.

In a broader analysis, it was also observed that the relationships between mA, PcFat, and PcFat variation in the second half of the activity were primarily influenced by the acceleration. However, in the case of women, an influence of PcFat on mA was detected, which may be explained by the presence of muscle pain or discomfort that may have influenced the movement patterns^2^ ^,^ ^3^. Future studies should address the presence of these symptoms.

Some limitations should be considered when interpreting the results of this research. First, sample bias is due to a lack of sample randomization. Hence, the randomization sampling process with a larger sample should be carried out in future studies. Second, IMU-related bias can reduce data accuracy. Measures have been taken to reduce it, but it would be interesting to use an IMU system to measure the orientation behavior of the upper limb and other kinematic features during ULPSIT.

According to our findings, incorporating upper limb acceleration measurement as part of a comprehensive monitoring strategy can be a valuable tool in managing and preventing excessive PcFat in the elderly, enhancing their performance, and optimizing interventions in clinical and sports settings.

## CONCLUSION

The acceleration behavior of the upper limb during a ULPSIT is linked to PcFat scores or its variation with differences between sexes. The PcFat scores, or its variation, were primarily influenced by the acceleration. However, in the case of women, an influence of PcFat on acceleration behavior was also detected.
